# How to facilitate the implementation of 3D models in China by applying good *in vitro* method practice for regulatory use

**DOI:** 10.3389/ftox.2023.1080528

**Published:** 2023-03-08

**Authors:** Yanfeng Liu, Zhenzi Cai, Nan Li, Nathalie Alépée

**Affiliations:** ^1^ L’Oréal Research & Innovation China, Shanghai, China; ^2^ L’Oréal Research & Innovation France, Aulnay-Sous-Bois, France

**Keywords:** good *in vitro* method practices, reconstructed human epidermis model, OECD test guideline 439, *in vitro*, EpiSkin^™^ skin irritation test, alternative

## Abstract

The Organization for Economic Co-operation and Development (OECD) Guidance Document No. 34 and No. 286 on Good *In Vitro* Method Practices (GIVIMPs) for the development and implementation of *in vitro* methods for regulatory use in human safety assessment have been endorsed. Considering that China is accelerating the development of alternative approaches in both research and acceptance, early application of these principles is beneficial to the implementation and acceptance of *in vitro* alternative methods in China. To promote the replacement of animal testing for regulatory use, L’Oréal initiated the EpiSkin™ skin irritation test (SIT) implementation program in China. More than 50 external scientists participated, and the method has been established in 34 organizations including authorities, industries, and testing service laboratories. Taking two collaborations with Guangdong CDC and Shanghai SGS for *in vitro* SIT as examples, we demonstrated a method implementation process in good alignment with the OECD principles. The current study illustrated the practical way in which both OECD Guidance documents assisted in the transfer and establishment of *in vitro* approaches and further promoted the future scientific recognition and acceptance of new OECD-accepted alternative testing methodologies in China.

## 1 Introduction

The Organization for Economic Co-operation and Development (OECD) as an intergovernmental organization co-ordinates and harmonizes policies to respond to international issues in the area of test validation and implementation. Practical guidance on principles and processes for the validation and acceptance of animal and non-animal test methods for regulatory hazard assessment purposes was developed and adopted. OECD Guidance Document (GD) No. 34 provides guidance on issues related to the validation of new or updated test methods and their related requirements for the acceptance of test methods for the hazard assessment (OECD, 2005). In addition, an OECD GD No. 286 on Good *In Vitro* Method Practices (GIVIMPs) for the development and implementation of *in vitro* methods for regulatory use in human safety assessment was recently endorsed (OECD, 2018). This guidance, initiated and led by the Joint Research Centre (JRC), primarily targets users who implement *in vitro* methods. GIVIMPs aims to reduce uncertainties in cell- and tissue-based *in vitro* method application but also provides guidance for *in vitro* method developers with the following objectives: 1) detailed update on good practices for *in vitro* method application; 2) help ensure the standard operating procedures (SOPs) are well-designed, robust, well-defined, and can be carried out in a Good Laboratory Practice (GLP) environment; 3) descriptions of the key aspects that may impact the reliability and relevance of the *in vitro* data; and 4) descriptions of the importance of reporting criteria, applying good experiment design, establishing acceptance criteria, and performance standards based on scientific evidence from the generated *in vitro* datasets.

GIVIMP incorporates the relevant elements of both GLP and Good Cell Culture Practice (GCCP) (Ulrey et al., 2021). As GLP mainly focuses on the reproducibility of non-clinical studies (OECD, 1998; Cooper-Hannan et al., 1999) and GCCP only provides reference for cells and tissues reproducible production (Coecke et al., 2005; Pamies et al., 2020), neither can serve as a comprehensive quality framework for the development or execution of *in vitro* methods. GIVIMP includes guidance on the execution of the entire test method and is intended to promote quality during an *in vitro* method’s life cycle (Ulrey et al., 2021).

Furthermore, both OECD documents on validation and on GIVIMP facilitate the application of the OECD Mutual Acceptance of Data (MAD) agreement for data generated by *in vitro* methods and to avoid any unnecessary additional testing (Eskes et al., 2017). Regarding to the MAD system, OECD countries and full adherents have agreed that a safety test carried out in accordance with the OECD Test Guidelines (TGs) and Principles of Good Laboratory Practice in one OECD country must be accepted by other OECD countries for assessment purposes. MAD is primarily intended to facilitate a harmonized approach and thereby avoid conflicting or duplicative national regulatory requirements. This saves the expense of duplicate testing for chemicals and eliminates the non-tariff trade barrier.

The OECD Test Guidelines for the testing of chemicals are a collection of the most relevant internationally agreed upon testing methods used by governments, industries, and independent laboratories to assess the safety of chemicals (OECD Website). The OECD TG 439 addresses the human health endpoint skin irritation based on the *in vitro* test system of the reconstructed human epidermis (RhE) model. This test guideline was originally adopted in 2010 including the EpiSkin™ and EpiDerm™ skin irritation test methods, updated subsequently to include additional test methods using other RhE models and the use of an alternative procedure to measure viability (OECD, 2021). According to the OECD GD, the adopted test method should be sufficiently robust and transferable among properly equipped laboratories with adequately trained staff before application (OECD, 2005). Laboratories should demonstrate the technical proficiency by using the 10 Proficiency Substances listed in OECD TG 439 prior to the routine use of the adopted methods. The use of both GD that are based on validated test methods promotes the generation of dependable data for human and animal health and environmental hazard assessment.

In China, regulations for toxicity testing of chemicals still rely primarily on animal testing methods. Yet, in recent years, significant progress has been seen in both research and industries for the promotion of alternative methods. China has participated in the International Cooperation on Alternative Test Methods (ICATM) as an observer since 2015 with an open mind to alternative methods in cosmetic safety assessment (Barroso et al., 2016). To date, five alternative methods are included in China’s cosmetics standards, and four methods have completed verification and are open for public opinion on the National Institutes for Food and Drug Control (NIFDC) website, covering skin irritation/corrosion, ocular toxicity, skin sensitization, phototoxicity, and genotoxicity (Luo et al., 2019). The 3T3 neutral red uptake (NRU) method for phototoxicity referring to TG 432 was accepted as cosmetic standards n° 147 (CFDA, 2016), and the transcutaneous electrical resistance (TER) assay for skin corrosion referring to TG 430 was accepted as cosmetic standards n° 136 (CFDA, 2017). China is accelerating the development and implementation of non-animal alternative methods, and many domestic research institutions and authorities’ laboratories have made further efforts to develop their knowledge and capability in the use of alternative methods (Yang et al., 2015; Barroso et al., 2016; Zhang et al., 2017).

Given the commercial availability of the EpiSkin™ RhE model in China, L’Oréal initiated the OECD TG 439 EpiSkin™ skin irritation test (SIT) method implementation program in China since 2011. More than 50 external scientists participated, and the method has been established in 34 organizations including authorities, industries, and testing service laboratories. Taking the collaboration with two laboratories, from SGS CSTC Standards Technical Services Co., Ltd. in Shanghai (SHSGS) and Guangdong Provincial Center for Disease Control and Prevention (GDCDC), for *in vitro* SIT as examples, here, we demonstrated the successful implementation process aligned with OECD GD No. 34 and No. 286 in China.

## 2 Materials and methods

### 2.1 Test system—EpiSkin™ reconstructed human epidermis model

The EpiSkin^™^ test system was purchased from Shanghai EPISKIN Biotechnology Co., Ltd (Shanghai, China). Consisting of fully differentiated basal layer, spinous layer, granular layer, and horny layer, the test system presents a properly multilayered and well-differentiated epidermis (Qiu et al., 2016). Its quality and reproducibility were ensured by the supplier, including barrier function and morphology analysis.

### 2.2 Main reagents

The test method protocol includes the listing and description of all preparations, reagents, supplies, and equipment needed, and all criteria and procedures for generating and evaluating test data. The reagents came from well-established sources, certified by the suppliers. The storage was performed according to the manufacturer’s specifications, meeting the OECD recommendations (e.g., GIVIMP, 4.2).

3-(4,5-Dimethylthiazol-2-yl)-2,5-diphenyltetrazolium bromide (MTT) and sodium dodecyl sulphate (SDS) were purchased from Sigma-Aldrich (St. Louis, MO, U.S.A.). Dulbecco’s phosphate buffered saline (D-PBS) with calcium and magnesium was obtained from Gibco (Grand Island, NY, U.S.A.). Acidic isopropanol was made of 0.04N HCl (Yonghua Chem, Shanghai, China) in isopropanol (Sigma-Aldrich).

### 2.3 EpiSkin™ skin irritation test method

The SOP is published on Database on Alternative Methods (DB-ALM) as protocol n° 131 (http://cidportal.jrc.ec.europa.eu/ftp/public/JRC-OpenData/EURL-ECVAM/datasets/DBALM/LATEST/online/DBALM_docs/).

Briefly, the tested chemicals, 10 μL of liquids or 10 mg of solids, were applied on the surface of the epidermis with triplicate tissues for 15 min of treatment at room temperature, followed by 42 h of post-incubation at 37°C, 5% CO_2_, and 95% humidity. Cell viability was determined by the MTT assay and measured optical density (OD) at 570 nm and expressed as a percentage normalized to the negative control set at 100%.

### 2.4 Method implementation process

The primary objective of this initiative was to assist the participants in gaining a practical understanding of the theory and application of the test method meeting OECD GD No. 34 requirement (2005). Moreover, the GIVIMP described the responsibility of *in vitro* method developers (GIVIMP 1.1) and clarified the integrality of formal training for quality assurance (GIVIMP 2.6). The current SOP was essential in a quality management system to ensure that the procedure was carried out in a consistent and reproducible way. The SOP (DB-ALM n° 131) publicly available describes clear work instructions for a trained user to minimize the risk for misinterpretation (GIVIMP 7.1, 7.2). Prior to the routine use of the OECD-adopted SIT method, the proficiency chemicals were evaluated by laboratories (*in vitro* method users) for training purposes to demonstrate the personnel’s ability to perform the method (GIVIMP 8.4).

Effectively in the current study, prior to the formal transferring phase, formal training experiments were performed by external participants from collaborating laboratories under supervision to properly ensure the SOP was carried out. The scientists (from L’Oréal, *in vitro* method developer) went to the collaborating laboratories (*in vitro* method users), helped to check the technical availability (equipment, reagent, and consumable…), and demonstrated the detailed operations. Then, participants performed the experiment under coaching. Two liquids (isopropanol, *in vivo* UN GHS No Cat., #9 in Table 1; cyclamen aldehyde, *in vivo* UN GHS Cat. 2, #4 in Table 1) and two solids (naphthalene acetic acid, *in vivo* UN GHS No Cat., #10 in Table 1; 1-methyl-3-phenyl-1-piperazine, *in vivo* UN GHS Cat. 2, #2 in Table 1) selected from OECD TG 439, representing both *in vivo* UN GHS No Cat. and Cat. 2, were proposed for experiments under supervision (P1).

**TABLE 1:** Characteristics and UN GHS classification of the 10 test chemicals.

No.	Chemical name	CAS #	Physical state	*In vivo* UN GHS classification	VRM classification	Purity	Supplier	Training (P1)
								Transfer (P2)
1	1-bromohexane	111-25-1	Liquid	Cat. 2	Cat. 2	98	Sigma-Aldrich	P2
2	1-Methyl-3-phenyl-1-piperazine	5271-27-2	Liquid	Cat. 2	Cat. 2	97	Sigma-Aldrich	P1, P2
3	Potassium hydroxide (5% aq.)	1310-58-3	Liquid	Cat. 2	Cat. 2	99.99	Sigma-Aldrich	P2
4	Cyclamen aldehyde	103-95-7	Liquid	Cat. 2	Cat. 2	90	Sigma-Aldrich	P1, P2
5	Heptanal	111-71-7	Liquid	Cat. 2	Cat. 2	95	Sigma-Aldrich	P2
6	Hexyl salicylate	6259-76-3	Liquid	No Cat. (*Optional* Cat. 3)	No Cat.	≥99	Sigma-Aldrich	P2
7	Heptyl butyrate	5870-93-9	Liquid	No Cat. (*Optional* Cat. 3)	No Cat.	≥98	Sigma-Aldrich	P2
8	Methyl stearate	112-61-8	Solid	No Cat	No Cat.	99	Sigma-Aldrich	P2
9	Isopropanol	67-63-0	Liquid	No Cat	No Cat.	≥99	Sigma-Aldrich	P1, P2
10	Naphthalene acetic acid	86-87-3	Solid	No Cat	No Cat.	≥95	Sigma-Aldrich	P1, P2

UN GHS classifications: No Cat., no category, non-irritant; Cat. 2, category 2, irritant.

According to the description in OECD TG 439, laboratories should also demonstrate technical proficiency by using the 10 Proficiency Substances (proficiency chemicals), prior to the routine use of the EpiSkin™ SIT method. Therefore, the formal transferring phase considering proficiency list chemicals (P2) with two runs of the qualified test was implemented by external participants individually, if needed, to fully transfer and establish the method in their laboratory. In case of unqualified run or tests, an additional third run of the test should be considered. For the transferring phase, participants independently ordered the tissue, scheduled the experiment, set up their study plan, and performed the test.

Here, we took two collaborations with SHSGS and GDCDC as examples, including experiments under supervision and the following transferring phase by independent operation, to demonstrate the method implementation process is in good alignment with GIVIMP.

For lab SHSGS, two participants first performed the experiments under supervision and then both implemented the test of 10 chemicals described in the OECD TG 439 proficiency list.

For lab GDCDC, one study director and one lab assistant first performed the experiments under supervision and then the lab assistant implemented the test of 10 chemicals described in the OECD TG 439 proficiency list.

### 2.5 Test chemicals

Chemical information including chemical name, Chemicals Abstract Service Registry Number (CASRN), *in vivo* UN GHS classification, validated reference methods (VRM) classification, physical state, purity, and supplier is shown in Table 1.

Ten chemicals (#1–#10) described in the OECD TG 439 proficiency list were used for independent transferring session (P2), and four of them (#2, #4, #9, and #10) were used for the experiment under supervision (P1).

### 2.6 Negative and positive controls

Control items were used to evaluate the proper performance of the test system and, therefore, the validity of the executed experiments (GIVIMP, 6.1). Negative control (NgC) was defined as an item for which the test system should not give an effect on the test system. Positive control (PC) was defined as an item for which there is an absolute grading of the response with a consistent and predictive effect on the test system (Rispin et al., 2006).

For each single test, tissue treated with NgC should exhibit OD reflecting the quality of the tissues that followed shipment, receipt steps, and all protocol processes and should not be outside historically established boundaries; and tissues treated with the PC should show mean cell viability within a historically established range, and correct responses to an irritant chemical under the conditions of the test method should be monitored.

In addition, the acceptance criteria of controls can be used to monitor correct responses, and results from controls can be compared with historical data to detect any drift in the assay system (Rispin et al., 2006). In the current study, according to the SOP of the method, NgC was D-PBS and PC was 5% SDS. Both NgC and PC substances were performed concurrently with test chemicals.

### 2.7 Acceptance criteria

Importantly, the acceptance criteria should be developed and detailed in the *in vitro* method SOP to demonstrate that the method performs consistently over time and between laboratories (GIVIMP 8.1). Currently, the EpiSkin^™^ SIT SOP described the quality acceptance criteria that are included in OECD TG 439 and shown in Table 2. The response and the variability of the NgC and the PC, and the variability of the test chemicals, were evaluated according to these criteria. The variability of standard deviation (SD) between tissue replicates of test chemicals and/or control substances should fall within 18%, indicating qualified result.

**TABLE 2:** Quality criteria defined in OECD TG 439.

Quality criteria	Acceptance
NgC: mean OD	0.6 ≤ OD ≤ 1.5
PC: mean viability	Via% ≤ 40%
Tissue variability (NgC, PC, test chemical)	SD ≤ 18%

In the transferring phase, all 10 chemicals were tested in at least two independent runs. When an invalid run occurred within the two runs, an additional third run was performed.

The reproducibility was determined by the following criteria:

Within-laboratory reproducibility (WLR) calculated as the percentage of chemicals that showed 100% consistent classifications between two valid runs.

Between-laboratory reproducibility (BLR) calculated as the percentage of chemicals that showed 100% consistent classification between two labs.

Laboratories should demonstrate technical proficiency through 10 chemicals described in OECD TG 439 and meet the criteria for predictive performance in TG 439 (80% sensitivity, 70% specificity, and 75% accuracy).

## 3 Results

### 3.1 Test system

The quality requirement on the test system is described in OECD GD No. 34 and No. 286, including test system characterization, extensive documentation on origins, and relevant safety information, e.g., GIVIMP 1.2.

The test system, EpiSkin^™^ test system, is a commercialized reconstructed skin model purchased from Shanghai EPISKIN Biotechnology Co., Ltd. (Shanghai, China). The skin model consists of a fully differentiated basal layer, spinous layer, granular layer, and horny layer. Its quality and reproducibility were ensured by the supplier with examination of morphology and barrier function.

Skin models were shipped in a special temperature-controlled environment (room temperature) monitored by the supplier, as recommended in GIVIMP 5.3. Also, the supplier also provided extensive documentation including origins and characterization of the test system, product usage, safety evaluation, quality assurance descriptions, safety practices for use and disposal of the test system, period of validity, cell culture maintenance, and transport condition.

**FIGURE 1:**
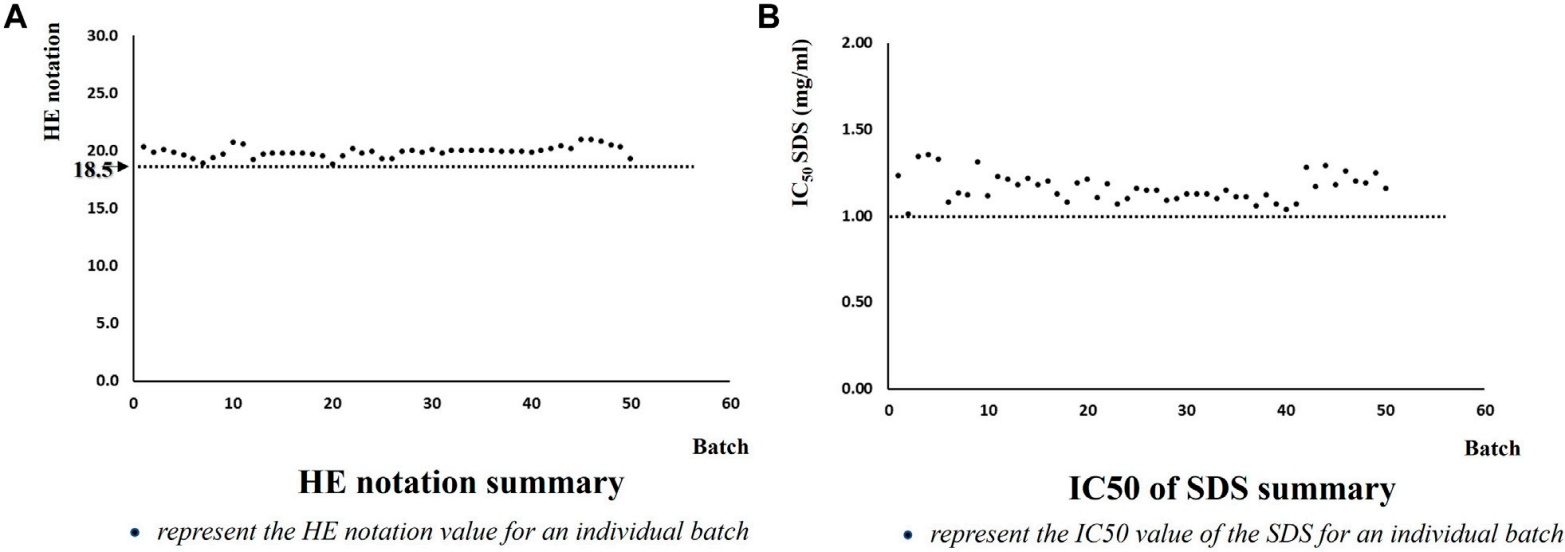
Historical QC trend chart.

The GIVIMP also recommends that the quality control chart should be used to routinely monitor the quality of the test system (GIVIMP 2.3). As described in TG 439, the quality control (QC) criteria include IC_50_, reflecting barrier function and morphology using histological examination (HE) notation. The acceptance range was established by the skin model supplier. The IC_50_ should be over 1.0, and the HE notation should be over 18.5. The QC trend chart provided by the supplier is shown in Figure 1. In up to 50 batches of skin model production, the data of HE notation (Figure 1A) and IC_50_ (Figure 1B) met the criteria, demonstrating good quality and reproducibility.

### 3.2 Negative and positive controls

According to OECD TG 439, the mean OD of the tissue triplicate treated with the negative control (D-PBS) should be ≥0.6 and ≤1.5, indicating good quality of the tissues after the test process. Mean cell viability of the tissue triplicate treated with the positive control (5% SDS), expressed as % of the negative control, should be <40%, indicating the correct response of the model to an irritant chemical.

**FIGURE 2:**
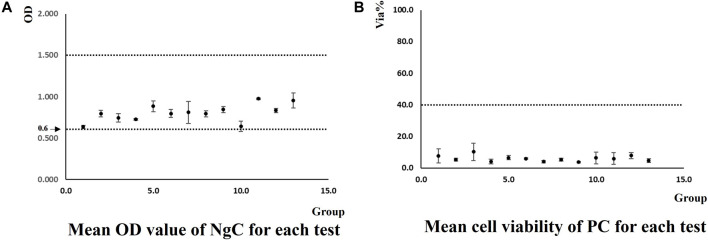
Mean OD of NgC and *via*% of PC generated by two laboratories in the method implementation process.

Two laboratories generated 13 groups of data for NgC and PC, as shown in Figures 2A, B, respectively. All obtained data met the acceptance criteria.

### 3.3 Cell viabilities and predictions for tested chemicals

#### 3.3.1 Experiments under supervision

**FIGURE 3:**
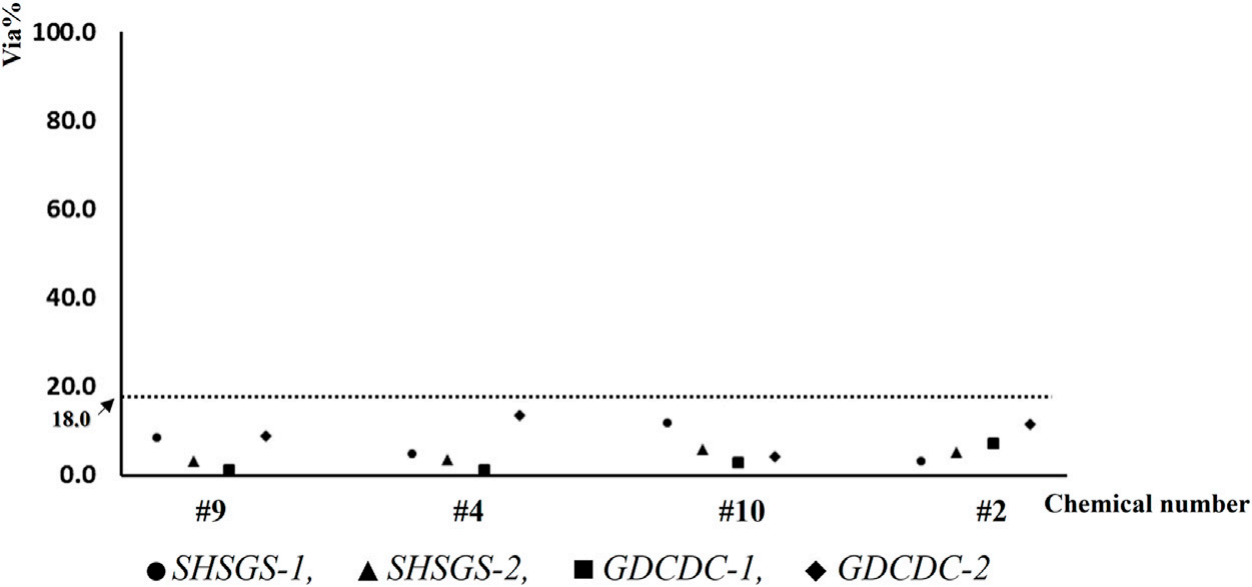
SD of cell viability for each run of test chemicals.

**FIGURE 4:**
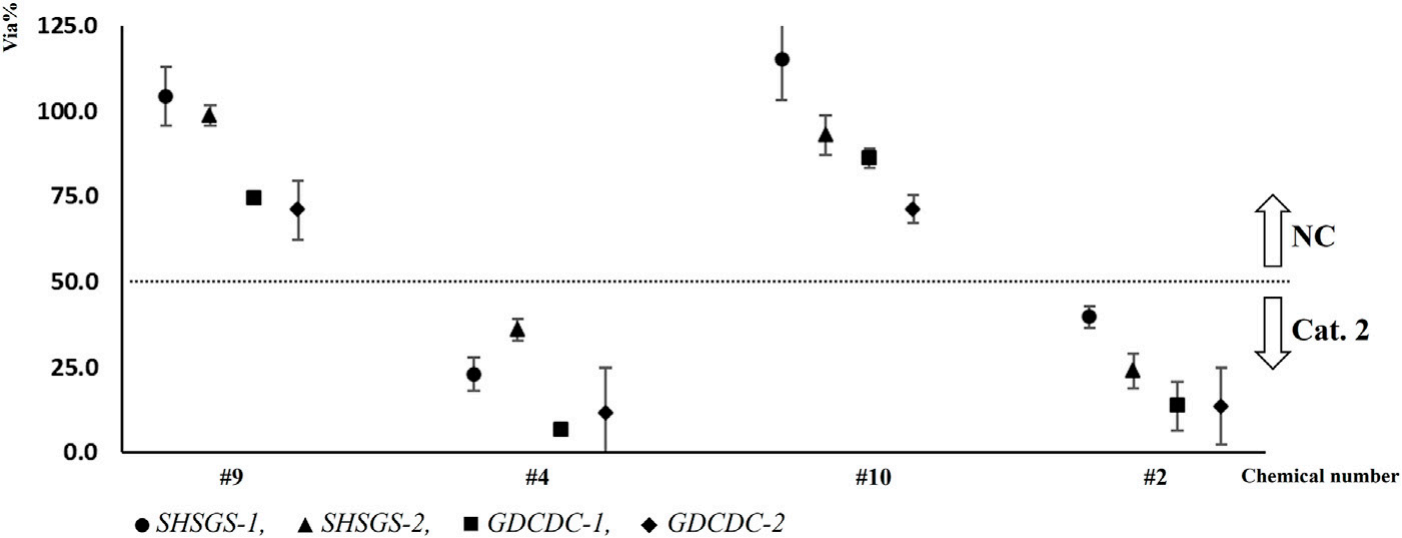
Distribution of mean cell viabilities for each run of test chemicals (Pl).

Two liquid and two solid chemicals (#9, #4, #10, #2) were set as P1 and used for experiment under supervision. SD of cell viability and the mean cell viability for each test are shown in Figures 3, 4, respectively.

Each participant from lab SHSGS and lab GDCDC obtained qualified results and correct predictions for all chemicals.

#### 3.3.2 Transferring phase

Ten reference chemicals (P2) described in the OECD TG 439 proficiency list were used for the transferring phase to investigate the transferability (reproducibility and predictability). At least two runs of the test were performed by two participants from lab SHSGS and one participant from lab GDCDC. The mean cell viability and predictions of each test chemical for all runs are shown in Table 3.

**TABLE 3:** Cell viabilities and predictions of all tested chemicals in two laboratories.

Nr	CAS #	*In vivo* UN GHS Cat.	VRM Cat.	Lab SHSGS—1 viability (%)			Lab SHSGS—2 viability (%)			Lab GDCDC viability (%)		
				Run	Mean ± SD	Prediction	Run	Mean ± SD	Prediction	Run	Mean ± SD	Prediction
1	111-25-1	Cat. 2	Cat. 2	1	8.4 ± 2.9	Cat. 2	1	47.9 ± 16.7	Cat. 2	1	32.6 ± 6.9	Cat. 2
				2	6.2 ± 1.3	Cat. 2	2	40.6 ± 3.8	Cat. 2	2	111.1 ± 13.5	No Cat.
				3	Not needed		3	Not needed		3	80.3 ± 14.2	No Cat.
2	5271-27-2	Cat. 2	Cat. 2	1	13.6 ± 7.1	Cat. 2	1	13.5 ± 11.3	Cat. 2	1	12.9 ± 7.0	Cat. 2
				2	18.5 ± 4.4	Cat. 2	2	18.7 ± 8.6	Cat. 2	2	8.6 ± 3.3	Cat. 2
3	1310-58-3 5%	Cat. 2	Cat. 2	1	4.6 ± 2.4	Cat. 2	1	7.5 ± 8.0	Cat. 2	1	2.5 ± 0.8	Cat. 2
				2	6.2 ± 1.1	Cat. 2	2	9.9 ± 5.9	Cat. 2	2	5.1 ± 0.8	Cat. 2
4	103-95-7	Cat. 2	Cat. 2	1		Cat. 2	1	11.6 ± 13.3	Cat. 2	1	17.9 ± 7.5	Cat. 2
				2	6.4 ± 1.1	Cat. 2	2		Cat. 2	2	24.9 ± 4.9	Cat. 2
				3	8.2 ± 4.0	Cat. 2	3	19.6 ± 8.3	Cat. 2	3	Not needed	
5	111-71-7	Cat. 2	Cat. 2	1	10.7 ± 5.1	Cat. 2	1	8.0 ± 1.7	Cat. 2	1	17.1 ± 4.6	Cat. 2
				2	8.1 ± 0.7	Cat. 2	2	6.6 ± 1.9	Cat. 2	2	13.6 ± 2.4	Cat. 2
6	6259-76-3	No Cat. (Optional Cat. 3)	No Cat.	1	82.4 ± 2.2	No Cat.	1	93.9 ± 7.4	No Cat.	1	95.5 ± 2.5	No Cat.
				2	91.5 ± 7.3	No Cat.	2	96.5 ± 14.6	No Cat.	2	98.7 ± 1.9	No Cat.
7	5870-93-9	No Cat. (Optional Cat. 3)	No Cat.	1	86.6 ± 2.6	No Cat.	1	105.8 ± 13.5	No Cat.	1	106.6 ± 7.1	No Cat.
				2	126.6 ± 1.1	No Cat.	2	126.0 ± 9.1	No Cat.	2	101.4 ± 5.9	No Cat.
8	112-61-8	No Cat.	No Cat.	1	79.6 ± 1.6	No Cat.	1	78.6 ± 5.4	No Cat.	1	94.3 ± 3.4	No Cat.
				2	104.9 ± 7.9	No Cat.	2	97.4 ± 6.8	No Cat.	2	101.8 ± 2.3	No Cat.
9	67-63-0	No Cat.	No Cat.	1	74.5 ± 1.2	No Cat.	1	71.0 ± 8.6	No Cat.	1	84.2 ± 7.6	No Cat.
				2	63.5 ± 1.1	No Cat.	2	70.9 ± 10.3	No Cat.	2	98.7 ± 3.0	No Cat.
10	86-87-3	No Cat.	No Cat.	1	86.1 ± 2.8	No Cat.	1	71.3 ± 4.0	No Cat.	1	91.7 ± 7.3	No Cat.
				2	92.3 ± 2.1	No Cat.	2	98.3 ± 4.1	No Cat.	2	100.1 ± 2.1	No Cat.

Over 63 tests were performed, and only two tests were unqualified (SD > 18%) for chemical #4 and two tests obtained mis-predictions for chemical #1, which represented a high reproducibility of 93.7% (59/63).

**FIGURE 5:**
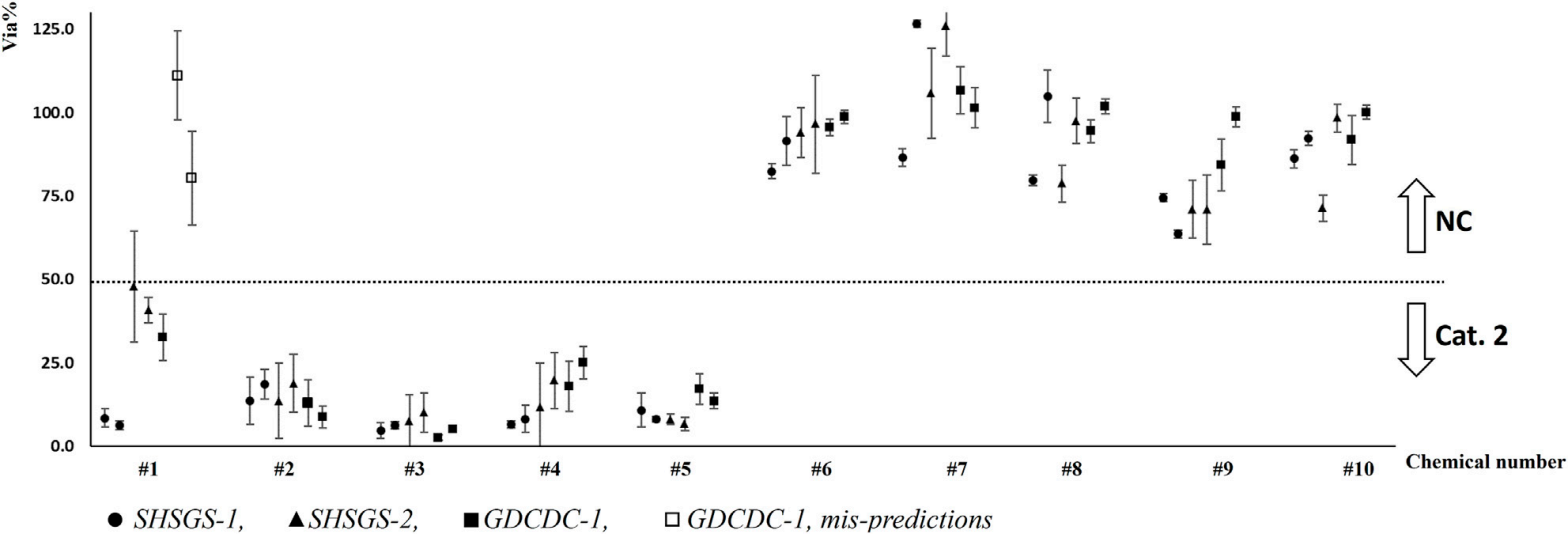
Distribution of mean cell viabilities of each qualified test for 10 chemicals in two laboratories (P2).

Considering lab SHSGS, the distribution of cell viabilities is shown in Figure 5. One unqualified test (SD > 18) was observed for each participant with #4 chemical (103-95-7). Mean cell viabilities are shown in Table 3 with strikethrough. Thus, the additional third run was performed for this chemical, and a qualified result was obtained. For chemical #1, participant 2 obtained a borderline result in the first run of test, although the run was still qualified and the chemical was correctly predicted, as shown in Table 3 with underline. The WLR was 100%, showing concordant prediction for two participants considering qualified tests.

Considering lab GDCDC, the distribution of cell viabilities is shown in Figure 5. One chemical (#1, 111-25-1) obtained misprediction in the second run as shown in Table 3 with *gray color*. Thus, an additional third run was performed in case there were any technical misoperations. However, the third run still showed misprediction on this chemical. Mean cell viabilities are shown in Table 3 with *gray color*. The WLR was 90%, showing nine out 10 chemicals obtained concordant prediction for two runs.

For both laboratories, technical support was provided throughout the process to align on operation details including equipment verification (incubator, balance, pipette, and spectrophotometer), proper application with exact volume and treatment period, proper washing step, post-incubation, MTT assay, and data analysis.

The BLR was 90%, with nine out of 10 chemicals obtaining concordant predictions for two laboratories.

If considering OECD TG 439, only one run of the test is sufficient for transferring. The current implementation with high reproducibility is very relevant.

### 3.4 Predictive performance

The predictive performance was evaluated according to descriptions in OECD TG 439, considering 10 chemicals tested in the transferring phase (P2).

The sensitivity may be defined as the ability of the method to detect the true positive rate, while the specificity is the ability of the method to correctly identify the true negative rate (OECD, 2005; 2018). Considering the current study, the sensitivity that No Cat. chemical predicted correctly was 100% (5/5) for lab SHSGS and 100% (5/5) for lab GDCDC; both met the criteria described in TG 439 (>80%). The specificity that Cat. 2 chemical predicted correctly was 100% (5/5) considering lab SHSGS and 86.6% (4.33/5) considering lab GDCDC; both met the criteria described in TG 439 (>70%). The accuracy that differentiates No Cat. and Cat. 2 chemical was 100% (10/10) considering lab SHSGS and 93.3% (9.33/10) considering lab GDCDC; both met the criteria described in TG 439 (>75%).

### 3.5 Comparison with historical data

**FIGURE 6:**
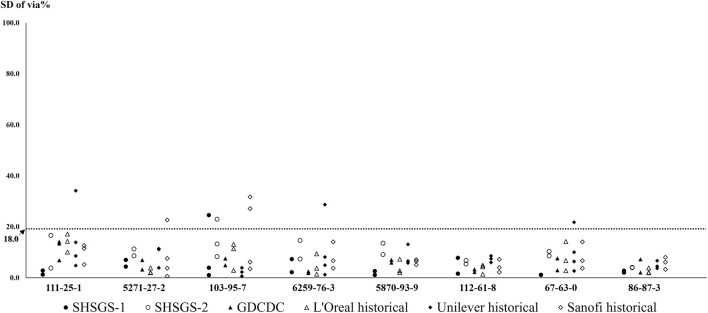
SD of viabilities for eight tested chemicals comparing with published historical data.

Compared the SD of viabilities for eight tested chemicals between the transferring phase and published historical data during the international validation study of EpiSkin™ SIT (Hoffmann, 2006), shown in Figure 6, the current study obtained good reproducibility. The formal training experiments and transferring phase allowed a correct and efficient transfer of the method.

## 4 Other activities—fast learning class

To meet the increasing need for the application and implementation of alternative methods in China, we also organized fast-learning classes to provide technical learning for extensive users including scientists, regulators, technicians, and anyone interested in this method. Forty-two participants with diverse backgrounds including authority laboratories, industrial companies, universities, research institutions, and testing service companies have participated in the fast-learning class since 2015. These small classes provided detailed coaching sessions strictly according to the SOP with two chemicals selected from P1 chemicals, including one liquid (#4, cyclamen aldehyde, *in vivo* Cat. 2) and one solid (#10, naphthalene acetic acid, *in vivo* No Cat.).

**FIGURE 7:**
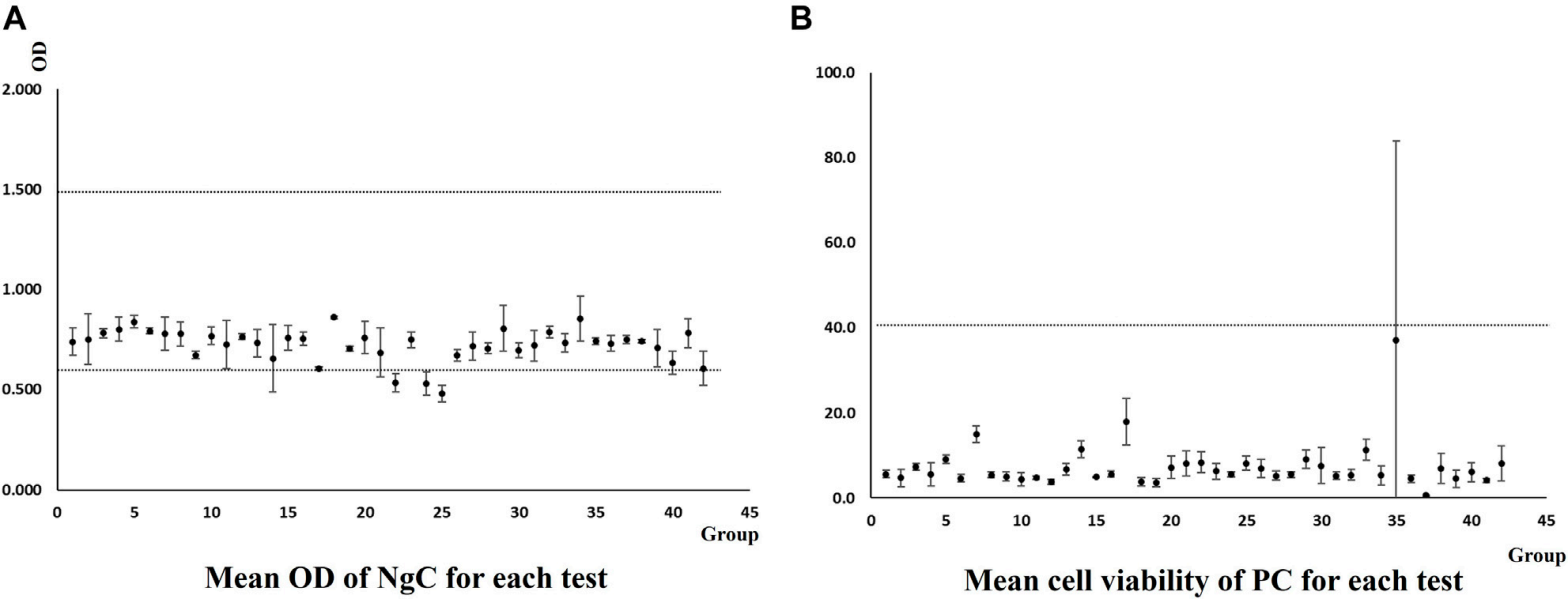
Mean OD of NgC and *via*% of PC generated in the fast-learning class.

With over 42 participants, 90.5% of runs were qualified, which demonstrates a successfully qualified study for 38 participants (shown in Figure 7). Among the studies, three NgC (OD < 0.6) and one PC (SD > 18) did not meet the acceptance criteria. This was due to technical issues, e.g., ungentle application or scratchy washing for NgC and the omission of the application for the unqualified PC, which were properly reported after the application and rinsing steps.

**FIGURE 8:**
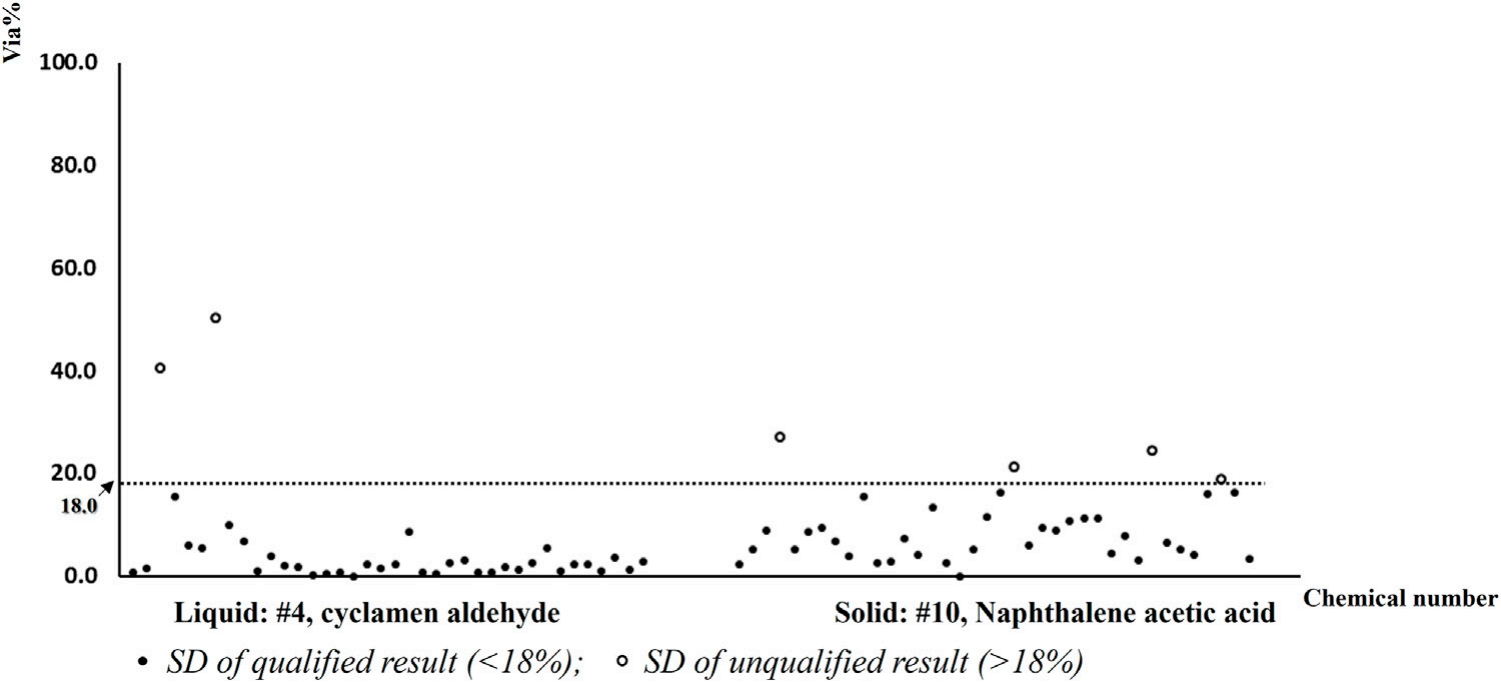
SD of cell viability for chemicals considering 38 qualified runs of test in the fast-learning classes.

**FIGURE 9:**
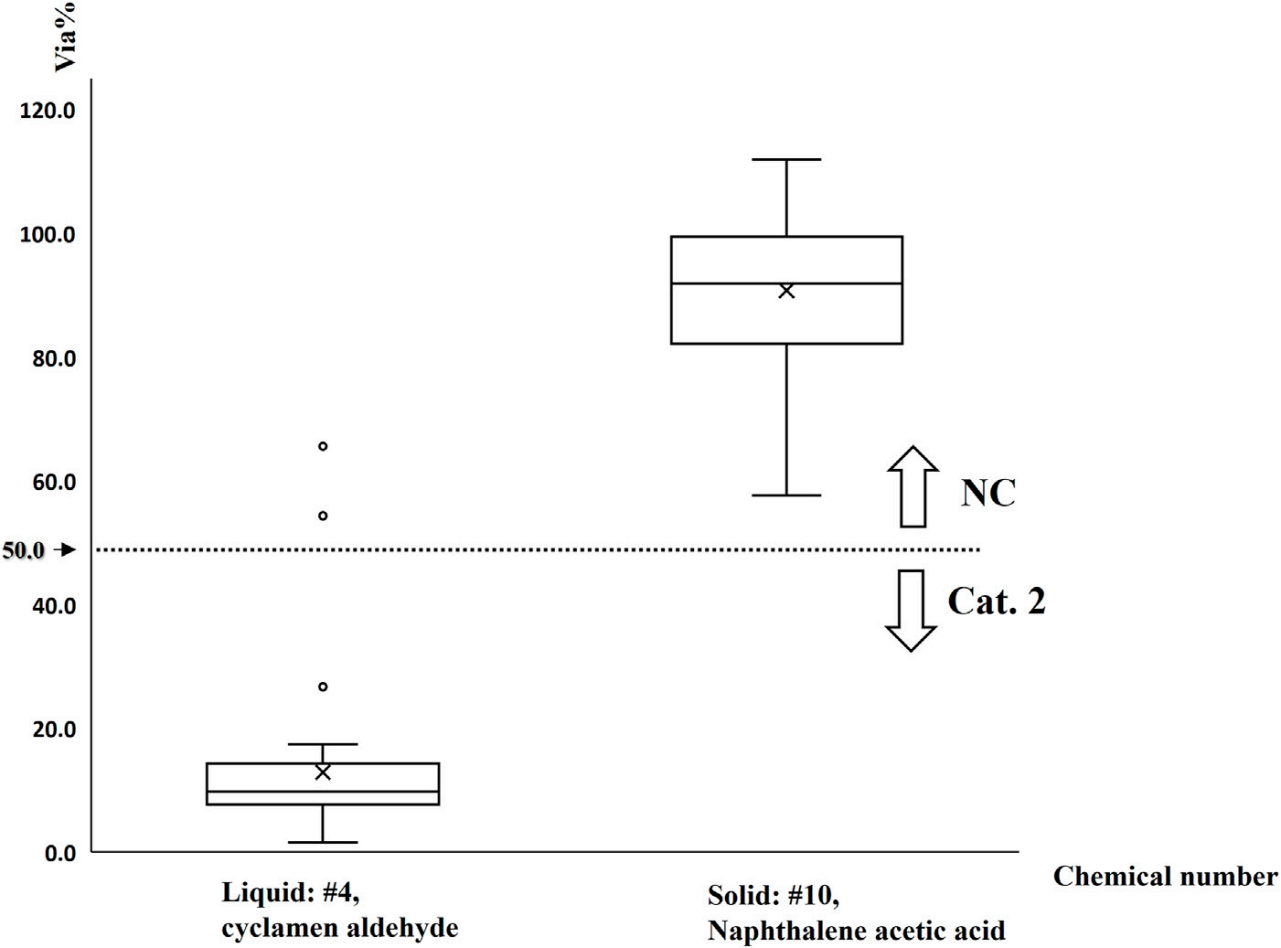
Distribution of mean cell viabilities of chemicals considering 38 qualified runs of test in fast-learning classes line within box: median; lower borderline of box: 25th quartile; upper borderline of box: 75th quartile; hollow dots: outside values.

Regarding the qualified results of 38 participants with the qualified control item, SD of cell viability for tested chemicals and the distribution of cell viabilities are shown in Figures 8, 9, respectively. For the liquid chemical (#4), two participants obtained unqualified results with higher SD (>18%, shown in Figure 8) also mispredicted. For the solid chemical (#10), four participants obtained unqualified results with higher SD (>18%, shown in Figure 8) while correctly predicted. The reproducibility was 92.1% (70/76) for qualified results with correct predictions.

## 5 Discussion

The OECD Guidance Document on GIVIMP was drafted for the development and implementation of *in vitro* methods for regulatory use in human safety assessment which is also equally applicable to non-guideline or not internationally recognized *in vitro* methods. The document provides guidance for developers and end users including 10 important aspects related to *in vitro* work, including responsibilities for key players within the life cycle of the *in vitro* method, quality considerations, facilities, materials and reagents, test system, control item and test item, SOP, performance, report and publishing, and records storage.

All these 10 aspects were considered both for the experiment under supervision and the transferring phase in the current study.

The test system is a commercialized skin model, and the supplier is a key player within the cycle of the *in vitro* method for ensuring the quality and reproducibility of the tissue. In this case, the supplier provides extensive documentation of their responsibilities, such as test system characterization. Aligning to the description for regional logistic, all the skin models were shipped in a special temperature-controlled environment (room temperature) monitored by the supplier. Also, all the tested chemicals were commercially available and purchased from certified suppliers. At first, the experiments were performed under supervision with scientists from L’Oréal strictly according to SOP. Subsequently, the second phase was conducted to demonstrate the technical proficiency of the participant laboratory. All record sheets and raw data were stored in safes with restricted access.

Regarding the EpiSkin™ SIT method, which is adopted in OECD TG 439 to identify No Cat. and Cat. 2 chemicals, the SOP (DB-ALM protocol n° 131) is published to specify details, instructions, and work sheets to ensure consistency and reproducibility.

The criteria for the control item and test item were defined in TG 439. If either NgC or PC included in a run fell outside of the accepted ranges, the run would be considered non-qualified and should be repeated. If SD of cell viabilities for the test chemical fell outside of the accepted range, this chemical should be retested. For lab SHSGS, both participants obtained higher SD for chemical #4 for one run; thus, an additional third run was performed. For lab GDCDC, SD for all tested chemicals met the criteria. Due to chemical #1 being mispredicted at the second run, an additional third run was performed in case of technical misoperations. Even though we realigned each step strictly according to the SOP, this chemical was still mispredicted. In the recent published skin models, misprediction and high variation (SD > 18%) were also observed in other skin models such as LabCyte Epi-Model24, Keraskin™, and EpiTRI (Kojima et al., 2014; Hwang et al., 2020; Liao et al., 2021). In addition, the latest version of TG 439 also updated the note that this chemical can have variable results in different laboratories, depending on suppliers.

For the current study, overall higher variation was observed for the fast-learning class than the supervised experiments together with the transferring phase. Unexpected misoperations occurred during the 1-vs-many coaching format (1 trainer vs. 2–3 participants) in the fast-learning class, such as missing application of chemical, scratching on the surface of the model using tips or cotton swab, improper rinsing, and other unaware reasons.

## 6 Recommendations

Although fast-learning classes enabled more participation of individuals from diverse backgrounds and provided a way to quickly understand the operational skills, full supervision and the complete transfer phase described previously are required to master the method. The formal training experiments under supervision together with the transferring phase, both strictly according to the SOP, ensured the participant’s operation under coaching, evaluated the transferability and reproducibility, and demonstrated a good example for the application of Good *in Vitro* Method Practices.

In conclusion, the EpiSkin™ SIT implementation program in China exemplifies the practical way in which the OECD GD No 34 and GIVIMP guidance can assist interested parties in the establishment of *in vitro* approaches. Although animal testing of the Draize test is primarily applied to regulatory toxicity testing of chemicals in China, the Draize skin test is criticized due to large variation and less reproducibility than the *in vitro* test method on the RhE model (Lee et al., 2017). The current program proves the high reproducibility and good transferability of the EpiSkin™ SIT method in various laboratories and helps convince the authorities to consider using 3D models as the standard tool to evaluate skin irritation. This constructive dialog among the test method developer, industries, and regulatory agencies staff should expedite the implementation and global acceptance of the OECD SIT 3D methods. This initiative paves the way toward future scientific recognition and acceptance of *in vitro* OECD-accepted testing methods (e.g., skin irritation and skin corrosion) and facilities the implementation of 3D models (e.g., epidermis and/or epithelium) in China.

## Data Availability

The original contributions presented in the study are included in the article/Supplementary Material; further inquiries can be directed to the corresponding author.

## Abbreviations

CASRN, Chemicals Abstract Service Registry Number; Cat. 2, Category 2; D-PBS. Dulbecco’s phosphate buffered saline; EURL ECVAM, European Union Reference Laboratory for Alternatives to Animal Testing; GDCDC, Guangdong Provincial Center for Disease Control and Prevention; GIVIMP, Good *In Vitro* Method Practice; GHS, globally harmonized system of classification and labeling of chemicals; HE, histological examination; IC_50_, half maximal inhibitory concentration; JRC, Joint Research Centre; MTT, 3-(4,5-dimethylthiazol-2-yl)-2,5-diphenyltetrazolium bromide; NgC, negative control; NIFDC, National Institutes for Food and Drug Control; No Cat., no category; NRU, neutral red uptake; OECD, Organization for Economic Co-operation and Development; PC, positive control; QC, quality control; RhE, reconstructed human epidermis; SD, standard deviation; SDS, sodium dodecyl sulphate; SHSGS, SGS CSTC Standards Technical Services Co., Ltd. in Shanghai; SIT, skin irritation test; SOPs, standard operating procedures; TER, transcutaneous electrical resistance; TG, Test Guideline; UN, United Nations; VRM, validated reference methods.
